# Cultural Identity Confusion and Psychopathology: A Mixed-Methods Study Among Refugees and Asylum Seekers in the Netherlands

**DOI:** 10.1097/NMD.0000000000000935

**Published:** 2019-02-04

**Authors:** Simon P.N. Groen, Annemiek J.M. Richters, Cornelis J. Laban, Jooske T. van Busschbach, Walter L.J.M. Devillé

**Affiliations:** * De Evenaar, North-Netherlands Centre for Transcultural Psychiatry, GGZ Drenthe Mental Health Institute, Beilen;; † Amsterdam Institute for Social Science Research, University of Amsterdam, Amsterdam;; ‡ University of Groningen, University Medical Centre Groningen, Rob Giel Research Centre, Groningen;; § Windesheim University of Applied Sciences, Zwolle;; ∥ University Medical Center Utrecht, Julius Center for Health Sciences and Primary Care, Utrecht; and; ¶ Advisory Centre on Migrants, Refugees, and Health (Pharos), Utrecht, the Netherlands.

**Keywords:** Postmigration stress, psychopathology, cultural identity, refugees and asylum seekers, trauma

## Abstract

Although there is ample empirical evidence that traumatic events, postmigration stress, and acculturation problems have a great impact on the mental health of refugees, so far no studies have included cultural identity after migration in the equation. This mixed-methods study conducted among Afghan and Iraqi refugee and asylum-seeker psychiatric patients aims to fill this gap. Associations between postmigration stress, symptoms of anxiety and depression disorders, and symptoms of posttraumatic stress disorder were significant. When differentiated for the two groups, associations with postmigration stress were no longer significant for Afghan patients, who were predominantly younger and more often single, lower educated, and without resident status compared with Iraqi patients. Qualitative results indicate that, in addition to psychopathology and postmigration stress, acculturation problems contribute to confusion of cultural identity. The findings suggest that reduction of postmigration stress and acculturation problems may clarify cultural identity and as such may contribute to posttraumatic recovery.

It is well-known that refugees and asylum seekers often have prolonged mental health problems. Several studies have shown that anxiety, depression, and posttraumatic stress disorder (PTSD) tend to be highly prevalent among these groups, even years after resettlement in the host country (Bogic et al., 2015). Earlier studies have focused on the relationship between experienced potentially traumatic events (PTEs) and psychopathology, whereas more recent studies have shown that postmigration living problems (PMLPs) among resettled refugees and asylum seekers have a significant impact on psychopathology as well (Carswell et al., 2011; Laban et al., 2005; Porter and Haslam, 2005).

Like all migrants, refugees and asylum seekers have to relate to norms and values in the host country that may be (partly) different from those in the home country. This process is referred to as acculturation, defined by Berry (2005, p 698) as “…the dual process of cultural and psychological change that takes place as a result of contact between two or more cultural groups and their individual members.” When this acculturation process is perceived as problematic, it is to be considered as a risk factor for mental illness of the respective migrant, along with biological and other psychological and social factors (Bhugra and Becker, 2005). More than other migrants, refugees and asylum seekers may be confronted with PTEs, PMLPs, and problematic acculturation as risk factors for mental health problems. These risk factors may have a troubling impact on cultural identity. Confusion regarding one's cultural identity, in its turn, may have negative consequences for mental health.

In a previous article, we defined cultural identity as the identity shaped by incorporated norms and values that constitute an image that an individual holds of himself or herself, which urges the individual to decide what is to be considered as right or wrong and what kind of behavior is appropriate or not, while these norms and values are negotiated within the (ethnic or ethno-religious) group to which the individual belongs (Groen et al., 2018). In a study by Mezzich et al. (2009), cultural identity was found to be relevant or even central for understanding mental health problems and levels of individual and social functioning in a culturally diverse patient population. Bhugra (2005), in a literature review on the relationship between migration, cultural identity, and mental health had already observed that when individuals migrate from one type of culture to another it is likely that they, depending on their personality traits, may develop psychiatric disorders.

We postulate that experiencing PTEs may have far-reaching consequences for an individual's inner core cultural values that shape thought and action, in other words, his or her cultural identity. The way in which PTEs and/or PMLPs may affect cultural identity has, however, so far remained unclear. To the best of our knowledge, this issue has not been studied until now. Given the complexity of the interrelationship between various risk factors for psychopathology in the population of our study and cultural identity, a qualitative approach may contribute to gaining a deeper insight. In our previous publication, cultural identity—subdivided into personal, ethnic, and social identity—was analyzed among 85 Afghan and Iraqi patients (Groen et al., 2018). Their personal life stories, their sense of belonging to an ethnic (minority) group, and their social embeddedness in the host society appeared to be strongly connected to both trauma-related stress and acculturation problems.

The aim of this study, conducted among newly referred Afghan and Iraqi patients, is to investigate interrelationships between cultural identity, aforementioned risk factors, and psychopathology. Associations between experienced PTEs, postmigration stress, and acculturation preferences are explored. Qualitative data were used to describe the process of the way these factors cohere with (changes in) cultural identity in the trauma-affected study population. Finally, by linking the results of both parts of the study, the implications of the study findings for mental health professionals in their clinical practice are addressed.

## METHODS

A concurrent design of quantitative and qualitative methods was applied in this study (Creswell and Zhang, 2009). Quantitative measures were used to explore patterns of associations between PTEs, PMLPs, acculturation, and psychopathology. In the same group of participants, qualitative data were collected and analyzed to gain an in-depth understanding of how these relationships may lead to mental health problems and may change cultural identity after migration. Results were disaggregated between Afghan and Iraqi participants to enhance insight in potential differences in outcomes between both groups.

### Participants

A consecutive sample of an eligible group of 100 Afghan and Iraqi patients was recruited from a Dutch Centre for Transcultural Psychiatry, between August 2012 and February 2015. The group of patients was part of a bigger case-control study aimed at including in total 100 patients and controls of both countries of origin together. The required size of the total sample aimed at studying a possible poor effect size (Cohen's *f*^2^) of 0.15 (power = 0.8; α = 0.05) in a multiple linear regression analysis with three predictors and three confounding variables (Cicchetti, 2008; Cohen et al., 2003; Soper, 2018). Invitation letters for the clinical assessment including an invitation for participation in the study were sent to all referred patients who were originally from Afghanistan or Iraq. Participants were selected from patients of these countries of origin, because they were the largest groups of refugees in the Netherlands and also the largest groups of patients in the center at the moment of inclusion. A second reason was to make the study population not too culturally heterogeneous while in the meantime avoid relying on results of one group only. Inclusion criteria were a minimum age of 17 years, at least resident in their home country until the age of 12, and speaking Dari, Arabic, Dutch, or English. Patients with florid psychosis or substance-related disorders, or those with cognitive disabilities, were excluded. Furthermore, patients who were fluent only in Pashto or any Kurdish language (Badini, Kurmanji, Sorani) were excluded, because translations of the questionnaires in these languages were not available. Two patients refused to participate because they expected the questionnaires to be too intrusive. Two were excluded because they had been referred to the center for a second opinion regarding diagnosis without the aim of being included in treatment; in this case, completion of the questionnaires would have been complicated. Four were excluded due to illiteracy and the unavailability of assistance in filling in the questionnaires. In one case, there was no Arabic interpreter available. The 91 potential remaining participants were informed about the study in the invitation letter that they received for their psychiatric assessment. After psychiatric assessment, they were given written and oral information about the purpose of the research, the guarantee of confidentiality, the procedures, the fact that participation was voluntary, and the right to withdraw without negative consequences for their treatment. Not all questionnaires were completed or returned after several attempts to stimulate completion. Eventually, 57 patients (response rate, 62.6%) were included in the study: 28 Afghan patients (response rate, 57.1%) and 29 Iraqi patients (response rate, 69.0%). The final sample of patients allowed us to study an effect size of 0.30 in a multiple linear regression analysis with three predictors and three confounding variables (Cicchetti, 2008; Cohen et al., 2003; Soper, 2018). Approval for the research study was granted by the University Medical Centre of the University of Groningen (Protocol ID 2012.404).

### Instruments

A structured questionnaire on sociodemographic characteristics covered the following items: sex, age, education, work, family situation, ethnic group, languages, importance of religion and politics, region of origin, juridical status, and length of stay in the Netherlands.

The Harvard Trauma Questionnaire (HTQ) was used to assess the number of PTEs (part 1) and the severity of trauma symptoms (part 2), and the Hopkins Symptoms Checklist–25 (HSCL-25) was used to assess the severity of anxiety and depression symptoms. Both are culturally validated (Mollica et al., 1992, 1987). The first part of the HTQ contains 20 items related to PTEs that participants may have experienced, witnessed, or heard about. Cronbach's α was 0.932 (Cronbach, 1951). The second part contains 30 items related to any trauma symptoms experienced 1 week before administration on a four-point Likert scale (1, not at all; 4, extremely). The score is calculated by dividing the sum of the results (from χ times 1 to χ times 4) by the number of questions (Cronbach's α = 0.969). The HSCL-25 contains 10 anxiety symptoms and 15 depression symptoms and was administered following the same procedure (Cronbach's α = 0.969).

Postmigration stress factors were gathered using the Postmigration Living Problems Checklist (PMLP-CL), which was adapted from Silove et al. (1997). This list includes 23 items and two open items on a four-point Likert scale (1, no; 4, very much), which could be identified by participants in the 12 months before administration (Cronbach's α = 0.875). In addition, participants were asked which three items worried them the most.

The Cortes-Rogler-Malgady Bicultural Scale (CRM-BS) was used to measure acculturation preferences. The CRM-BS was found feasible by Latino, Korean, and Chinese psychiatric patients in a New York City hospital: 64.5% perceived the questionnaire to be very easy to somewhat easy. Test-retest reliability showed Pearson's *r* of 0.82 for the total scale. Ratios in a discriminant validity test of the first 10 and second 20 items were 1.70 (SD = 1.75) for the multiethnic sample (validated in Mezzich et al., 2009). In accordance with CRM-BS instructions, participants were asked to refer to their experiences up to 12 months before administration on a four-point Likert scale (0, not at all; 4, very much). For our purposes, the name of the host country (United States) and language (English) were changed to “the Netherlands” and “Dutch,” respectively. The CRM-BS contains 20 items, the first 10 for ethnic group (“origin”) and the remaining 10 for Dutch culture (“host”); the former correspond to the latter. Before responding to the items, participants could indicate their ethnic group to ensure that they would refer to, for example, being Kurdish or Iraqi, Hazara, or Afghan. Results were analyzed by dividing the scale of “origin” by the scale of “host” (range, 0–60) for the total research population (Cronbach's α = 0.850) and for Afghans and Iraqis separately. To calculate cutoff scores, subscales of the CRM-BS were made for “origin” (Cronbach's α = 0.892) and “host” (Cronbach's α = 0.824) and scores of origin divided by host of less than 1.00 were considered to display a preference for norms and values of the host society.

All questionnaires were translated into Arabic, Dari, and Dutch by official native language interpreters and were controlled by cointerpreters of the Free University Language Centre in Amsterdam. Separate sets of questionnaires were composited for Afghan and Iraqi participants, including Dutch and Dari or Dutch and Arabic versions.

Qualitative data were collected using the Brief Cultural Interview (BCI, Groen et al., 2017) from all participants who agreed to participate and who had received the aforementioned questionnaires. The BCI is an operationalization of the Outline for Cultural Formulation in the fourth edition of the *Diagnostic and Statistical Manual for Mental Disorders* (*DSM-IV*, American Psychiatric Association, 1994) and covers the following components: cultural identity of the individual; cultural explanations of the individual's illness; cultural factors related to the psychosocial environment and levels of functioning; and cultural elements in the relationship between the individual and the clinician (*cf*. Lewis-Fernández, 1996). Twenty-three of the interviews were conducted in Dutch, 16 in Dari, 1 in Pashto, 12 in Arabic, 2 in Turkmen, 1 in Assyrian, 1 in Sorani, and 1 in English; an interpreter by phone was arranged when necessary. There were no differences between Afghan and Iraqi participants regarding the need to use an interpreter: 17 (60.7%) of 28 and 17 (58.6%) of 29, respectively.

### Data Analysis

Data from the prestructured questionnaires were analyzed using the Statistical Package for Social Science for Windows, version 23. Descriptive analyses were used to describe the characteristics of the sample, with χ^2^ and independent samples *t*-tests to test differences between Afghan and Iraqi participants. Participants were divided into younger and older groups according to the median age of 30 years. Associations between the various instruments in the total sample were studied by Pearson correlation. Linear regression analysis was performed to explore the relationship between anxiety/depression and PTSD symptoms with PTEs, acculturation, and PMLPs. This analysis was first done for the whole group controlled for age, sex, and juridical status. To gain insight into differences in the associations between the two groups, the analyses were also done for Afghan and Iraqi participants separately. Imputation of the mean was performed for missing values (maximum = 2 per scale).

Ad hoc reconstructed interview reports of the BCIs were analyzed using ATLAS.ti, version 7.0 (ATLAS.ti Scientific Software Development GmbH, Berlin), which is based on a grounded theory approach (Glaser and Strauss, 1967). Narrative reconstructions from the BCI reports were first open coded, to identify the same parameters as in the quantitative analysis. When fragments from the reports were recognized as conforming to a previously identified code, this particular text was assigned to the existing code. Codes were accumulated in a code list until the point of saturation was reached.

## RESULTS

### Sociodemographic Characteristics

The study group of 28 Afghan and 29 Iraqi participants (see Table 1) consisted of more men than women (65% *vs*. 35%), with a larger sex difference among the Afghans (71% *vs*. 29%) compared with the Iraqis (59% *vs*. 41%). There was a significant difference between both groups in terms of age, marital and juridical status, and length of stay in the Netherlands: the Afghan participants were much younger, more often single, and more often asylum seekers than the Iraqis, and their duration of residence in the Netherlands was less than half that of the Iraqis. Ethnic variety among both groups was extensive, with ethnic minorities dominating. The level of education received in the country of origin and current employment status in the host country varied significantly between the two groups: Afghan participants were more often uneducated or lowly educated and unemployed than Iraqi participants. Satisfaction with the employment situation among those employed was low in both groups (only 15% was satisfied).

**TABLE 1:**
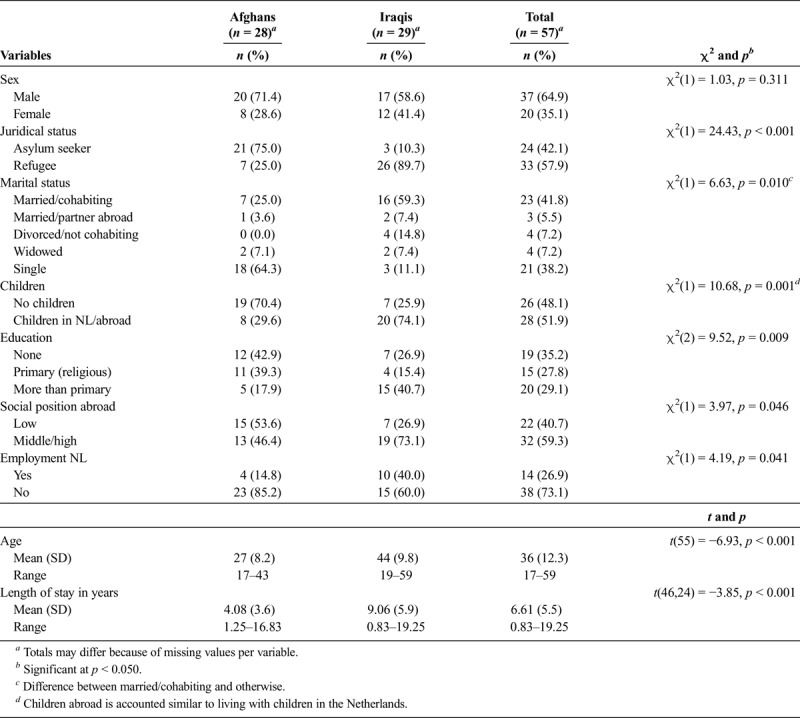
Sociodemographic Characteristics of the Study Population of Afghan and Iraqi Participants (*n* = 57)

### Psychopathology, PTEs, PMLPs, and Acculturation: Descriptions and Associations

Levels of PTSD and anxiety/depression were relatively high in the total study population, and were higher among the Afghan than Iraqi participants (see Table 2). The study population had experienced a mean of almost 10 PTEs per person, Afghans significantly more than Iraqis. The most frequently mentioned experienced PTEs were ill health without medical care, imprisonment, lack of food and water, being close to death, and having been threatened with physical torture. The most frequently mentioned PMLPs were missing the family, insecurity about the future, loneliness, health problems, and fear of being sent back. Acculturation scores were a little lower than 1, meaning that all of the participants attached almost equal appreciation of Dutch items and items of their ethnic group, as included in the CRM-BS, with a slight preference for Dutch items. There was no significant difference between Afghans and Iraqis.

**TABLE 2:**
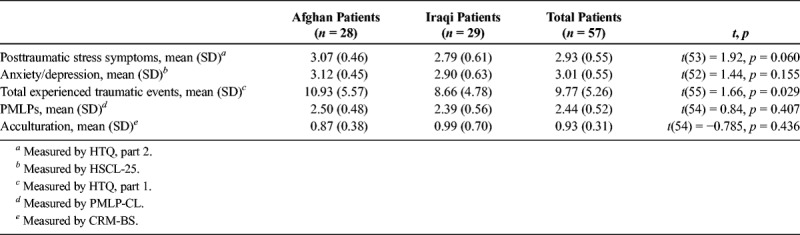
PTSD, Anxiety/Depression Disorder, Number of Experienced Traumatic Events, PMLPs, and Acculturation in Afghan and Iraqi Participants (*n* = 57)

For PTSD and anxiety/depression, no differences were found between men and women, or between participants who were 30 years or younger and those older than 30 years. Results show a significant difference between men and women and between younger and older participants with regard to the number of PTEs. Men had experienced more PTEs than women (10.73, SD = 5.51 *vs*. 6.70, SD = 4.57; *t*(55) = 2.79, *p* = 0.007), and younger participants had experienced more PTEs than older participants (12.43, SD = 4.56 *vs*. 7.21, SD = 5.13; *t*(55) = 3.95, *p* = 0.000). No such differences were found for PMLPs and acculturation preferences.

For the whole study sample, a significant correlation was found between all three measures: anxiety/depression, PTSD symptoms, and PMLPs (Table 3). For Afghan participants, PMLPs were no longer significantly associated, but anxiety/depression symptoms remained significantly associated with PTSD symptoms. For Iraqi participants, however, PMLPs remained significantly correlated to both anxiety/depression and PTSD symptoms. There was no significant correlation between acculturation or PTEs and any other measure.

**TABLE 3:**
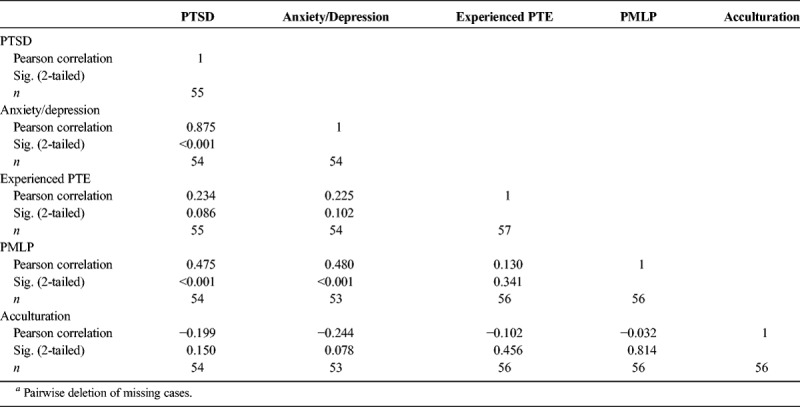
Correlations Between PTSD Symptoms, Anxiety/Depression Symptoms, Experienced PTEs, PMLPs, and Acculturation in Afghan and Iraqi Participants (*n* = 57)*^a^*

Multivariate regression analyses for PTSD and for anxiety/depression were carried out. Stepwise regression, both backward and forward, resulted in the exclusion of all variables except for PMLPs. The number of PTEs and PMLPs, acculturation, age, sex, and juridical status were entered into a forced model. Only PMLPs remained significant both for PTSD and anxiety/depression. For both models, the Cohen's effect sizes were of a medium level, respectively, 0.41 and 0.40 (Cicchetti, 2008; Table 4). When both groups were analyzed separately, neither PMLPs nor any other variables remained significant for Afghans.

**TABLE 4:**
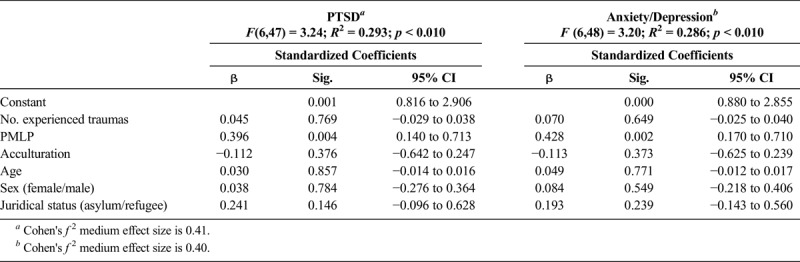
Multivariate Linear Regression of PTSD and Anxiety/Depression Symptoms

### Findings From the Interviews

#### Trauma and Ethnicity-Related Vulnerability

In the interviews, belonging to an ethnic or ethno-religious minority appeared to be a high-risk factor for experiencing PTEs. When participants did not belong to such a minority, other sociodemographic characteristics distinguished them from mainstream groups in their country of origin, such as growing up in a communist family, working for the government, or working for a United States company or the US army. PTEs, as experienced by participants in both groups, were culturally specific, particularly among those belonging to ethnic minorities. Among Afghans, this included violence against Shiites in Herat, a Hazara being used as a slave by the Taliban, a man being kidnapped because his wife worked for a foreign company, mothers being threatened by force to give away a daughter for marriage, and so on. The following quote reveals the living conditions of a single 21-year-old Hazara participant from Afghanistan, who had been used as a slave by the Taliban at the age of 12 after both of his parents died shortly one after the other:

“…there were a lot of Taliban over there. The young boys of 12 and 13 years old, they just took them, and then just raped them. Those people [from the village where he lived, SG] just had to give them money or food. They had nothing to eat, but they had to give them, for example, a sheep” (Interview in Dutch).

#### Postmigration Stress

Postmigration stress factors did not fully correspond with factor items of the PMLPs checklist used in the quantitative part of the study, but there is a considerable overlap. Codes for postmigration stress were grouped into the following: being without family; disappointments; fear; and language problems. The most recurrent item was being without family, both in the home country and in the country of residence, and encompassed the following codes: living without family; being away from family; no contact with children in the home country; missing the family; worries about the family in the home country; mental health problems within the family; responsibility for the family; marriage problems; and acculturation differences within the family. Participants often mentioned homesickness and loneliness as a means to express their strong wish to be with their family, and a lack of social contacts as a means to express being unable to discuss problems within the family. Indirectly, the urge to follow news about their country of origin could be linked to concerns about their family, but not in all cases.

Disappointments concerned the way of living, education, and work in the host society. Relevant interview fragments were coded as follows: living in the Netherlands; afraid of expectations in the Netherlands; imprisonment episodes; homelessness; adaptation problems; problems at work; socioeconomic status; lack of work; lack of education; lack of activities; financial problems; problems with institutions; problems with law and/or police; failing legal obligations; and travel difficulties. Failing legal obligations referred to problems experienced in the obligatory civic integration course that follows the obtaining of legal status in the Netherlands or problems obtaining a Dutch passport. Apart from being arrested and placed in custody as a foreigner, problems with the law and/or police also concerned disappointments over the Dutch legal system; some of the participants had difficulties accepting lower punishments for crimes committed against them in the Netherlands when compared with punishments in their home country.

Participants expressed fear in terms of being found crazy, being sent back, in relation to the asylum procedure, and threats from or in the home country. Fear of being found crazy often recurred in interviews as the fear that the mental condition would further deteriorate to a point that would be shameful, taboo, and stigmatizing. Fear of threats from or in the home country referred to harm that could come to relatives who had been left behind. This was found among those who were on a black list in their country of origin; they still feared that they could be discovered and caught.

Language problems referred to misunderstandings, difficulties reading letters, being dependent upon others for translation, limited abilities to communicate with Dutch people, and not being able to travel autonomously. Constrained Dutch language capabilities led to situations of not understanding or misunderstandings. Many participants complained about not being able to acquire the Dutch language despite their desire to speak it, which was expressed in terms of a structural living problem.

Postmigration stress was different for Afghan compared with Iraqi participants. Problems related to the asylum procedure were predominant in interviews with Afghans. Fear of being sent back only appeared in interviews with Iraqis who experienced problems in the asylum procedure. Afghans mentioned loneliness more often than Iraqis. Lack of social support was dominant among Afghans, as a married 22-year-old Hazara male participant from Afghanistan stated:

“…I do not know anyone there [in the Dutch village where he lived, SG]. Yeah, for example, a friend, or anyone who helps me, let us say. I know two or three friends, let us say, in school, but they are in Assen [a city some 12 kilometers away from the village where he lived, SG]. There, where I live, …there is nothing over there. It is a small village, I think for an old man” (Interview in Dutch).

Marriage problems were dominant in interviews with Iraqi participants. Missing family members and problems of not being with family members who were left behind in the home country were themes among Iraqi participants exclusively, as well as acculturation differences with family members and adaptation problems. Worrying and news about (family in) the home country, failing legal obligations, problems with institutions, and problems with the law and/or police were dominant among Iraqi participants.

Among participants in this study, postmigration stress factors appear to raise doubts about the questions “Who am I?” and “Whom do I belong to?” Being physically without family provoked such a strong feeling of psychological connectedness to the family in the home country that participants seem to be left with the perception that part of them has been bereft. Disappointments about their way of living in the host country refer to dissatisfaction with participants' current sense of self and their meaning in society. Fear as experienced by participants designates insecurity about the self and as problematic experienced emotional distance to the other. Language problems limit ways of communication, expressing oneself, and perspectives on exchanging norms and values.

### Acculturation

Interview questions concerning acculturation generated the following codes: feeling understood and perceived cultural differences. Participants often declared that they did not feel well or only sometimes understood, both in general and by clinicians in particular. Many Afghans were more negative than positive about feeling understood, or they had mixed feelings. Iraqis were slightly more positive, although a majority still held mixed feelings. In both groups, residential status did not influence the level of feeling understood. Cultural differences were mostly conceived as being large. Some outspoken participants described them as “differences between heaven and hell” in favor of the host society. Feeling safe, freedom, and respect were the most frequently mentioned positive aspects. Some participants specifically declared that they were no longer afraid to go out on the streets, in comparison to in their home country. This was the case in particular for threatened ethnic minorities such as Afghan Hazara and Iraqi Christians.

The most troublesome cultural difference compared with the Dutch was being far away from family. This is similar to the PMLP code not being with family, but here participants distinguished themselves from Dutch people who were close to their family. The absence of “warm” social contacts in combination with the self-directedness of people in the host country was another frequent acculturation issue. Participants' personal situation in the present and their experiences in the past had a strong influence on the perception of cultural differences. One 25-year-old participant who had experienced problems in Afghanistan because he is a Shiite Hazara and his wife had worked for a foreign company stated:

“What strikes me here is the tranquility. That people do not interfere with one another. That someone is free. Those are the things that are not there in Afghanistan…. Here, no one interferes with your religion. It is not like Muslims who interfere with everything. Now I am here, my wife knows I am here. She easily goes to a doctor alone. I am not afraid that something will happen to her. The freedom, the freedom, that people allow each other their freedom” (Interview with a Dari interpreter).

The main dilemmas that most participants faced, from an acculturation point of view, was of finding themselves in a much safer place than in their home society, yet experiencing social losses and having to struggle to participate in society. The gain of more freedom to speak and act was challenged by the absence of family members and worries about them. Acculturation difficulties seem to confuse confidence related to participation in the host society, a sense of belonging, and levels of functioning. Mixed feelings about whether or not being understood in the host society threaten participants' feelings of belonging and create feelings of alienation. Perceived cultural differences point in the same direction, but in this case, in comparison to people in the host society who live in a family setting and to warm social contacts in the past compared with the present.

### Cultural Identity

Effects on cultural identity of having experienced PTEs, suffering from postmigration stress, and acculturation difficulties could be identified on a personal, ethnic, and social level. Personal identity characteristics such as age, sex, marital status, education, work, and social class or position appeared to influence the likelihood of experiencing traumas and acculturation problems, or of suffering from postmigration stress. For example, in Afghanistan under Taliban rule, it is considered culturally unacceptable for young women to engage in premarital relationships, receive education in English, or work for a state company. Such sex restrictions could lead to personal identification and acculturation problems in the Netherlands when their Dutch counterparts fail to understand these norms and values. In Iraq, women (especially married women) who experienced sexual abuse could suffer from culture-related shame due to the loss of family honor. Postmigration stress related to family issues could permeate personal identity, in the sense of feeling lonely or worthless: “being without family is having no life at all” or “I am safe, but without family.” Difficulties in identifying with Dutch peers due to cultural differences equally interfered with feelings of worthlessness, powerlessness, and being unappreciated on a personal level.

Ethnicity played a major role in the risk of exposure to trauma and, to a lesser degree, postmigration stress and acculturation problems. Violence against ethnic and other minority groups intervened at the ethnic identity level, especially for Hazara in Afghanistan, and Kurds and Christian minorities in Iraq. Participants often referred to the history of ethnic violence in their country of origin to give meaning to the traumatic events they had experienced. The most frequently mentioned postmigration stress was being an ethnic minority, fear for the safety of one's family, as well as loneliness and lack of social contacts due to living in an area where members of one's ethnic group are few. The acculturation process was complicated by ethnicity through the avoidance of social contacts with people not of their own ethnic group, and feelings of constrained acceptance and integration.

Social identity aspects such as family in the host or home country, social role or position in the family, social status, social relations, partner relationships, and social contacts were expressed in the context of postmigration stress and acculturation problems more than for experienced traumas. Participants mostly referred to postmigration stress in terms of feeling socially detached, worries about the family, or feeling guilty toward the family. Acculturation problems often denoted cultural differences in social interactions between the “cold” (host) and “warm” (home) society, acculturation problems among family members, differences in acculturation between generations, or the accusation of westernizing too much made by other Afghans or Iraqis. Participants explained that in both Afghanistan and Iraq, cultural demands require that the eldest son or daughter in a family where the father or mother has been killed should leave school and find work; however, this caused a higher risk for exposure to traumatic events because of the lack of parental protection.

In conclusion, the many sociocultural changes after traumatic experiences and postmigration stress result in far-reaching changes in cultural identity on all three levels—personal, ethnic, and social—as the two following vignettes show.

Vignette 1

Twenty-year-old Afghan Sahar (a pseudonym) was diagnosed with PTSD, depression, and suicidal ideations. Sahar had experienced traumatic events after her refusal to accept a forced marriage to a Sunni man, being Shiite herself, and secretly having a Shiite boyfriend. Members of the Sunni family of the man whom Sahar was supposed to marry murdered her boyfriend and raped her in front of her family. Afterwards they also threatened to kill her father, so Sahar and her family fled. In the Netherlands, she attempted suicide by cutting her wrists. At the time of the research, none of the family members held residency status, and all lived in a center for asylum seekers. The conclusion from the interview was that the traumatic events were deeply rooted in a local culture where a girl is not supposed to refuse a proposed marriage. Sahar's refusal violated the honor of the Sunni family, who in turn sought revenge by killing the man she wanted to marry and brutally violating her and thus her family's honor. This led to such a degree of shame that, according to the cultural code, the raped girl would have to take her own life to restore the violated honor of the family, something that Sahar attempted. Fear in her case was deeply rooted in the risk of being kidnapped and forced into marriage, which was anchored in her cultural identity as the eldest daughter (social identity) of a Shiite minority (ethnic identity) who displayed culturally inappropriate behavior related to sex, age, and premarital status (personal identity). As a consequence, Sahar felt worthless and guilty in relation to her family, in particular toward her father who suffered from heart problems. She experienced difficulties acquiring the Dutch language, being partly illiterate and uneducated (postmigration stress). And she felt too much shame to enter into social contact with other Afghans, let alone with Dutch peers (acculturation problems).
  In this example, Sahar's personal identity as a young woman in Afghanistan who was supposed to marry—and indeed should not refuse a forced marriage—was conceived as problematic and unclear, and was especially troubled by her Shiite ethnic identity and complicated by her social identity as the eldest daughter who is supposed to be a role model.

Vignette 2

A 50-year-old man from Iraq, Salam (a pseudonym), was diagnosed with PTSD after being kidnapped in Syria, where he was visiting an ill sister, 3 years after his initial arrival in the Netherlands. He had been imprisoned for 4 or 5 months in Syria, where he had been mistreated. With the help of another sister, Salam had managed to return to the Netherlands. However, because he had been absent for a long time, his social welfare allowance had been stopped. A neighboring young man frequently started fires in his garden, causing fear in Salam. During the interview, Salam revealed that he was Sabi Mendaean, a discriminated and suppressed Christian-related minority that holds John the Baptist as its prophet. He used to have an active social life during the good times back in Iraq, but in the Netherlands, language barriers hampered his social contacts with the Dutch. Furthermore, in the city where he lived peers from his ethno-religious group were rare, therefore he desired to move to another city where other Sabi Mendaeans lived. He felt lonely and useless because he had not been able to work for 5 years, and acquiring the Dutch language was difficult. His current mental health problems were found to have an impact on his pretraumatic cultural identity as a middle-aged socially active and economically successful father (personal and social identity) from a Christian-related minority (ethnic identity). The consequences of migration were a lack of social contacts with ethnic peers, loneliness, financial problems, and language barriers (postmigration stress), and feeling useless, powerless, and unable to participate in Dutch society (acculturation problems).
  In this second example, expectations of personal identity (what a middle-aged man from Iraq is supposed to do) and social identity (being economically successful and socially active) as well as ethnic identity (as a Sabi Mendaean) were troubled in the postmigration process of a man who was unable to work, had financial problems, and who lacked social contacts, especially with his ethno-religious peers.

## DISCUSSION

The quantitative findings of this study clearly point to a persistent association of postmigration problems with trauma-related psychopathology among 57 Afghan and Iraqi participants, whereas the number of experienced traumas is not significantly associated with psychopathology. Studies of PMLPs among refugee and asylum-seeker populations not in treatment have already shown their impact on psychopathology (Laban et al., 2005; Porter and Haslam, 2005); this study concerns psychiatric patients with PTSD and/or anxiety/depression who have been referred to a mental health institute. Even in this group, which experienced a mean of almost ten PTEs, current postmigration stress seems more important for psychopathology than experienced traumas. Apparently, resources for coping with stress fall short after resettlement. The participants in this study seem unable to regain material, psychosocial, financial, and other resources after experiencing adversities. In the conservation of resources theory, the loss of valuable resources enhances stress, whereas striving to retain, protect, and build these and new resources stimulates stress recovery (Hobfoll, 1989). The findings of this study among traumatized refugees and asylum seekers support this theory.

When differentiating between both groups of patients, the association of PMLPs with psychopathology becomes insignificant for Afghan participants, while it remains significant for Iraqis. Given the sociodemographic characteristics of both groups, this is a remarkable finding. It could be expected that mostly young, single, and low-educated Afghans who are mainly asylum seekers would be more vulnerable to PMLPs than married and middle to highly educated Iraqis with residency security. However, in this study, this is not the case. Length of stay in the host country and escalating violence in the country of origin appear to reinforce the association of PMLPs with psychopathology among Iraqi participants. The mean duration of residency of Iraqis in this study is more than twice that of the Afghan participants. The former are unemployed or working below their education level and are more often dissatisfied with their current employment than Afghans, although the employment rate of the latter group is even lower. Although it has been shown that length of stay among asylum seekers may lead to an increase in mental health problems (Laban et al., 2005; Uribe Guajardo et al., 2016), dissatisfaction about one's current socioeconomic situation, resulting in feelings of uselessness, powerlessness, and increased worthlessness, may contribute to mental health problems among refugees as well. During the research period, the threat of the Islamic State in some parts of Iraq was tremendous and dominated the world news. Fears about the fate of family members left in the home country have been found to be a high-risk factor for psychopathology (Nickerson et al., 2010), and indeed it is one of the most prevalent PMLPs among both groups in this study.

The findings of this study show that acculturation preferences have no significant association with psychopathology. Mean scores for both groups indicate that they have a slight preference for host (Dutch) norms and values over those of their home country, and the interview data reveal a more or less balanced representation of values attached to cultural differences between host country and country of origin. In the interviews, the acculturation process appears to lead to acculturation problems, which in turn influence coping with stress. Uncertainty over being understood and the cultural dilemmas that trouble participants appear to be in line with other, quantitative, studies that conclude that acculturation problems may lead to an increase in mental health problems (Ince et al., 2014). People may find comfort in more freedom of choice and speech compared with their home country, but experience discomfort related to a lack of warmth in social contacts and embeddedness in the host country. In this study, participants experience some level of discomfort in a setting in which they have mixed feelings over being (mis)understood, which lead to questions about who they had been in the past, who they are now, and who they will be in the future. Moreover, most acculturation studies focus on migrants who potentially have sufficient abilities for successful participation in society and the maintenance of their cultural heritage and identity (Fassaert et al., 2011), whereas the participants in this study are constrained by their mental health problems that undermine these abilities. This finding implies that in conjunction with trauma treatment, the resilience of refugee patients should be strengthened through guidance in the acculturation process.

According to the interviews, traumatic experiences, trauma-related psychopathology, postmigration stress, and acculturation problems appear to confuse cultural identity among this study group. Trauma seems to be deeply interwoven into the personal, ethnic, and social self. Norms and values that constitute an image of the self, and deciding between right and wrong, all of which give direction to every thought and action, touch a deeper level of the self than postmigration stress. In this study, it was found that the context of one's personal life story, including sociodemographic variability, could inform clinicians in terms of how the experience of traumatic events or postmigration stress in particular may have caused the patient to question his or her cultural orientation, which originally would have been self-evident. The relevance of cultural differences in personal identity for PTSD is not limited to Afghanistan or Iraq (*e.g.*, Jobson and O'Kearney, 2008). A focus on ethnic identity offers the opportunity to differentiate within a culture and to increase awareness of the vulnerability of certain ethnic minorities in countries such as Afghanistan and Iraq in terms of experiencing traumatic events. Ethnic minorities from other countries often are more vulnerable than mainstream ethnic groups for these experiences, as well as has been shown in a Somalian case study (Groen, 2009). The disproportionate representation of ethnic and ethno-religious minorities in the study population and deviation otherwise from the majority of the population in the home country is an indication in that direction. When clinicians include the sociocultural context into their treatment sessions, opportunities will arise for a better understanding of role responsibilities within the family that may change after experiencing traumatic events, and even more of consequences of the transition from an interdependent to an independent society (Bhugra, 2005; Taylor and Usborne, 2010).

Forced migration additionally includes cultural bereavement (Bhugra and Becker, 2005), in terms of losses such as being detached from family ties, a lack of meaningfulness in society (work, social status), and being unable to manifest oneself in society (language, education). Postmigration stress intensifies these losses, as the study population is confronted with loneliness, worries about family members in the home country, and experiences of disappointment in the host society. When a clinician succeeds in improving the patient's coping with postmigration stress, cultural identity may become clearer than before and a more positive condition for trauma treatment may be created.

This study suggests further research including a control group of participants from the same countries of origin who do not receive treatment in a mental health institute. They would most probably have less postmigration stress, fewer acculturation problems, and therefore, a clearer cultural identity. Whether the mental health problems of refugee patients, which disturb their sense of self to some degree already, reinforce acculturation problems remains to be studied further through comparison with such a “healthy” control study group. The findings of this study indicate that cultural identity confusion might be a barrier to recovery from mental health problems and coping well with PMLPs. Therefore a controlled study is recommended, focusing on the effects of offering a meaningful life fulfillment, such as (voluntary) work or project activities, in terms of helping refugee patients to recover from trauma-related disorders.

The findings of this study have to be interpreted with some caution. First, the relatively low number of participants limits the generalizability of the outcomes of the quantitative analysis. Taking the scores in some of the analyses into account, associations between acculturation preferences and psychopathology might have become significant if more participants had been included. Second, the sampling of the research population within the restricted research period led to a certain sociodemographic imbalance between Afghan and Iraqi patients, especially in terms of age, marital status, education, and juridical position. Reasons for a higher nonresponse rate among Afghan patients were hard to track down. Often those not wanting to participate refused to give a reason for not participating. We suspect that a high level of shame toward having mental health problems played a role. Further, because most Afghan patients were asylum seekers, they may have been reluctant to participate because of not wanting to endanger their asylum procedure. Although differences in sociodemographic characteristics did not influence PMLPs or acculturation outcomes, if the composition of both groups had been more similar, the quantitative findings might have been easier to interpret. Third, there might be methodological shortcomings in the quantitative analyses, because all participants reported high scores on the HSCL-25 and the HTQ, which may have led to a “restriction of range.”

## CONCLUSIONS AND RECOMMENDATIONS

The increasing influx of refugee and asylum seeker patients will have an effect on mental health care, due to psychopathology resulting from premigration and postmigration stress among these patients. This study indicates that postmigration stress among refugees and asylum seekers not only remains significantly associated with psychopathology after controlling for sociodemographic variables and acculturation, but also suggests a far-reaching effect on cultural identity as the carrier of deep-rooted norms and values. Getting a solid, secure position in the host society as a firm basis for recovery from mental health problems is complicated by being torn between safety and freedom on the one hand, and facing difficulties in adapting to a different sociocultural order of norms and values on the other. Equally, being released from life-threatening stress, but remaining strongly attached to—yet separated from—a sense of deeply rooted meaningfulness (through family, work), is another complication. The findings of this study imply that giving attention by health professionals to (changes in) cultural identity may lead to a better understanding of trauma-related psychopathology and so enhances opportunities for adequate treatment. This implication applies also to refugees from other countries of origin than Afghanistan and Iraq.

Based on our study findings, we recommend that:

- both researchers and clinicians take into consideration that postmigration stress and acculturation problems may confuse cultural identity
- both researchers and clinicians bear in mind that fears about the fate of one's family in the home country (*e.g.*, due to war and IS actions) is an important postmigration stressor
- clinicians take into consideration that cultural identity confusion might be a barrier to recovery from mental health problems and adequate coping with postmigration stress
- the exploration of changes and confusion in cultural identity will become common use in the clinical assessment and practice with refugees and asylum seekers
- the use of the Cultural Formulation as this is helpful in assessing cultural factors affecting the clinical encounter, such as cultural identity, and enhances culturally sensitive diagnosis and care
- clinicians, including psychotherapists, strengthen, in conjunction with trauma treatment, the resilience of refugee patients through guidance in the acculturation process
- research will be conducted among nonpatient groups to explore differences in cultural identity confusion between patients and nonpatients
- an increased recognition of the value of mix-methods research and an increased implementation of this kind of research, because the combination of qualitative and quantitative methods enhances insight in complicated processes such as the one addressed in this article.

## References

[bib1] American Psychiatric Association (1994) *Diagnostic and statistical manual for mental disorders* (4th ed). Washington: American Psychiatric Association.

[bib2] Berry JW (2005) Acculturation: Living successfully in two cultures. *Int J Intercult Relat*. 29:697–712.

[bib3] Bhugra D (2005) Cultural identities and cultural congruency: A new model for evaluating mental distress in immigrants. *Acta Psychiatr Scand*. 111:84–93. 15667427 10.1111/j.1600-0447.2004.00454.x

[bib4] Bhugra D Becker MA (2005) Migration, cultural bereavement and cultural identity. *World Psychiatry*. 4:18–24. 16633496 PMC1414713

[bib5] Bogic M Njoku A Priebe S (2015) Long-term mental health of war-refugees: A systematic literature review. *BMC Int Health Human Rights*. 15:29–70. 10.1186/s12914-015-0064-9 PMC4624599 26510473

[bib6] Carswell K Blackburn P Barker C (2011) The relationship between trauma, post-migration problems and the psychological well-being of refugees and asylum seekers. *Int J Soc Psychiatry*. 57:107–119. 21343209 10.1177/0020764009105699

[bib7] Cicchetti DV (2008) From Bayes to the just noticeable differences to effect sizes: A note to understanding the clinical and statistical significance of oenologic research findings. *JWE*. 3:185–193.

[bib8] Cohen J Cohen P West SG Aiken LS (2003) *Applied multiple regression/correlation analysis for the behavorial sciences* (3rd ed). Mahwah, NJ: Lawrence Earlbaum Associates.

[bib9] Creswell JW Zhang W (2009) The application of mixed methods designs to trauma research. *J Traumatic Stress*. 22:612–621. 10.1002/jts.20479 19960518

[bib10] Cronbach LJ (1951) Coefficient alpha and the internal structure of tests. *Psychometrika*. 16:297–334.

[bib11] Fassaert T De Wit MA Tuinebreijer WC Knipscheer JW Verhoeff AP Beekman AT Dekker J (2011) Acculturation and psychological distress among non-Western Muslim migrants—a population-based survey. *Int J Soc Psychiatr*. 57:132–143. 10.1177/0020764009103647 19933252

[bib12] Glaser BG Strauss AL (1967) *Discovery of grounded theory. Strategies for Qualitative Research*. Chicago, IL: Aldine.

[bib13] Groen S (2009) Recognizing cultural identity in mental health care: Rethinking the Cultural Formulation of a Somali patient. *Transcult Psychiatry*. 46:451–462. 19837781 10.1177/1363461509343087

[bib14] Groen SPN Richters JM Laban CJ Devillé WLJM (2017) Implementation of the Cultural Formulation through a newly developed Brief Cultural Interview: Pilot data from the Netherlands. *Transcult Psychiatry*. 54:3–22. 28157446 10.1177/1363461516678342

[bib15] Groen SPN Richters JM Laban CJ Devillé WLJM (2018) Cultural Identity Among Afghan and Iraqi Traumatized Refugees: Towards a Conceptual Framework for Mental Health Care Professionals. *Cult Med Psychiatry*. 42:69–91. 28108844 10.1007/s11013-016-9514-7 PMC5842267

[bib16] Hobfoll SE (1989) Conservation of resources: A new attempt at conceptualizing stress. *Am Psychol*. 44:513–524. 2648906 10.1037//0003-066x.44.3.513

[bib17] Ince BU Fassaert T De Wit MA Cuijpers P Smit J Ruwaard J Riper H (2014) The relationship between acculturation strategies and depressive and anxiety disorders in Turkish migrants in the Netherlands. *BMC Psychiatry*. 14:252–263. 25189615 10.1186/s12888-014-0252-5 PMC4172911

[bib18] Jobson L O'Kearney R (2008) Cultural differences in personal identity in post-traumatic stress disorder. *Br J Clin Psychol*. 47:95–109. 17708833 10.1348/014466507X235953

[bib19] Laban CJ Gernaat HB Komproe IH Van der Tweel I De Jong JT (2005) Postmigration living problems and common psychiatric disorders in Iraqi asylum seekers in the Netherlands. *J Nerv Ment Dis*. 193:825–832. 16319706 10.1097/01.nmd.0000188977.44657.1d

[bib20] Lewis-Fernández R (1996) Cultural formulation of psychiatric diagnosis. *Cult Med Psychiatry*. 20:133–144. 8853962 10.1007/BF00115858

[bib21] Mezzich JE Ruiperez MA Yoon G Liu J Zapata-Vega MI (2009) Measuring cultural identity: Validation of a modified Cortes, Rogler and Malgady Bicultural Scale in three ethnic groups in New York. *Cult Med Psychiatry*. 33:451–472. 19543817 10.1007/s11013-009-9142-6

[bib22] Mollica RF Caspi-Yavin Y Bollini P Truong T Tor S Lavelle J (1992) The Harvard Trauma Questionnaire. Validating a cross-cultural instrument for measuring torture, trauma, and posttraumatic stress disorder in Indochinese refugees. *J Nerv Mental Dis*. 180:111–116. 1737972

[bib23] Mollica RF Wyshak G Marneffe de D Khuon F Lavelle J (1987) Indochinese versions of the Hopkins Symptom Checklist-25: A screening instrument for the psychiatric care of refugees. *Am J Psychiatry*. 144:497–500. 3565621 10.1176/ajp.144.4.497

[bib24] Nickerson A Bryant RA Steel Z Silove D Brooks R (2010) The impact of fear for family on mental health in a resettled Iraqi refugee community. *J Psychiatr Res*. 44:229–235. 19875131 10.1016/j.jpsychires.2009.08.006

[bib25] Porter M Haslam N (2005) Predisplacement and postdisplacement factors associated with mental health of refugees and internally displaced persons: A meta-analysis. *JAMA*. 294:602–612. 16077055 10.1001/jama.294.5.602

[bib26] Silove D Sinnerbrink I Field A Manicavasagar V Steel Z (1997) Anxiety, depression and PTSD in asylum-seekers: Associations with pre-migration trauma and post-migration stressors. *Br J Psychiatry*. 170:351–357. 9246254 10.1192/bjp.170.4.351

[bib27] Soper DS (2018) A-priori sample size calculator for multiple regression [software]. Available at: http://www.danielsoper.com/statcalc.

[bib28] Taylor DM Usborne E (2010) When I know who "we" are, I can be "me": The primary role of cultural identity clarity for psychological well-being. *Transcult Psychiatry*. 47:93–111. 20511254 10.1177/1363461510364569

[bib29] Uribe Guajardo MG Slewa-Younan S Smith M Eagar S Stone G (2016) Psychological distress is influenced by length of stay in resettled Iraqi refugees in Australia. *Int J Ment Health Syst*. 10:4. 26793271 10.1186/s13033-016-0036-z PMC4719335

